# Expiratory central airway collapse – an overlooked entity? Two case reports

**DOI:** 10.1097/MD.0000000000022449

**Published:** 2020-10-16

**Authors:** Piotr Janowiak, Katarzyna Rogoza, Alicja Siemińska, Ewa Jassem

**Affiliations:** Department of Pneumonology and Allergology, Medical University of Gdańsk, Mariana Smoluchowskiego 17 street, 80-214, Gdańsk.

**Keywords:** asthma, chronic obstructive pulmonary disease, expiratory central airway collapse, excessive dynamic airway collapse, tracheobronchomalacia

## Abstract

**Introduction:**

Expiratory central airway collapse is defined by excessive inward bulging of either tracheobronchial posterior membrane or cartilage. The former is called excessive dynamic airway collapse (EDAC), and the latter, depending on the site of collapse, tracheomalacia, bronchomalacia or tracheobronchomalacia. Due to their non-specific symptoms and lack of awareness amongst clinicians they tend to be mislabeled as common obstructive lung disorders, or complicate their course undetected. Particular controversies refer to EDAC sometimes considered just as a symptom of obstructive lung disease and not a separate entity. Nonetheless, a growing body of evidence indicates that EDAC might be present in patients without apparent obstructive lung disease or it might be an independent risk factor in chronic obstructive pulmonary disease or asthma patients.

**Patient concerns:**

Patient #1 was admitted because of idiopathic chronic cough whereas patient #2 was admitted for differential diagnosis of dyspnea of uncertain etiology. In both patients symptoms were unresponsive to bronchodilators and inhaled corticosteroids.

**Findings and diagnosis:**

In both patients an excess collapse of tracheobronchial posterior membrane was detected during bronchoscopy; in patient #1, of right main bronchus and right upper lobe bronchus and in patient #2 of right upper lobe bronchus and both main bronchi. Excess central airway collapse in patient #2 was also visualized on expiratory chest CT. In patient #1 spirometry did not reveal obturation, whereas in patient #2 only mild, irreversible, obstruction was revealed, disproportionate to patients significant breathlessness.

**Interventions:**

Both patients were treated with N-acetylcysteine and adjustable positive expiratory pressure valves.

**Outcomes:**

Due to aforementioned treatment chronic cough in patient #1 subsided almost completely whereas patient's #2 dyspnea improved significantly.

**Conclusions:**

In presented cases EDAC was an unexpected finding, even though, it firmly corresponded with reported symptoms. Treatment modification led to improvement of patients quality of life.

## 1 Introduction

Expiratory central airway collapse (ECAC) is a term including 2 pathologically and clinically separate entities: excessive dynamic airway collapse (EDAC) and malacia of tracheobronchial cartilage. The latter is named according to the exact location of collapse that is, respectively, tracheomalacia, bronchomalacia or tracheobronchomalacia (TBM). The primary TBM is often a part of a congenital syndrome, whereas secondary causes include airway trauma, medical procedures, such as tracheostomy, chronic recurrent airway infections, chronic external compression of central airways by for example, enlarged lymph nodes, autoimmune inflammation, or mechanical factors, for example, following pneumonectomy.^[1,2]^ EDAC is defined by excessive inward bulging of tracheobronchial posterior membrane. However, its status as a separate pathological entity has been questioned.^[3]^ Sceptics argue that significant collapse of central airways posterior membrane during exhalation accompany obstructive lung diseases due to decreased transmural pressure caused by increased airway resistance and decreased elastic recoil.^[3]^ Significant bulging of tracheobronchial posterior membrane should then warrant investigation for obstructive lung diseases, such as chronic obstructive pulmonary disease (COPD) or asthma.^[1,3]^ However this does not always seem the case. A study of 6 military personnel, diagnosed for exertional dyspnea with expiratory wheezing, despite extensive differential diagnosis provided no proof for causative role of obstructive lung disease.^[4]^ Indeed, expiratory collapse of tracheobronchial posterior membrane, shown in expiratory computer tomography (CT) and bronchoscopy, suggested that EDAC might have been the only cause of exertional dyspnea^[4]^

Herein, we present 2 cases supporting the notion that some EDACs may be considered an independent entity.

### 1.1 Case #1

**Figure 1:**
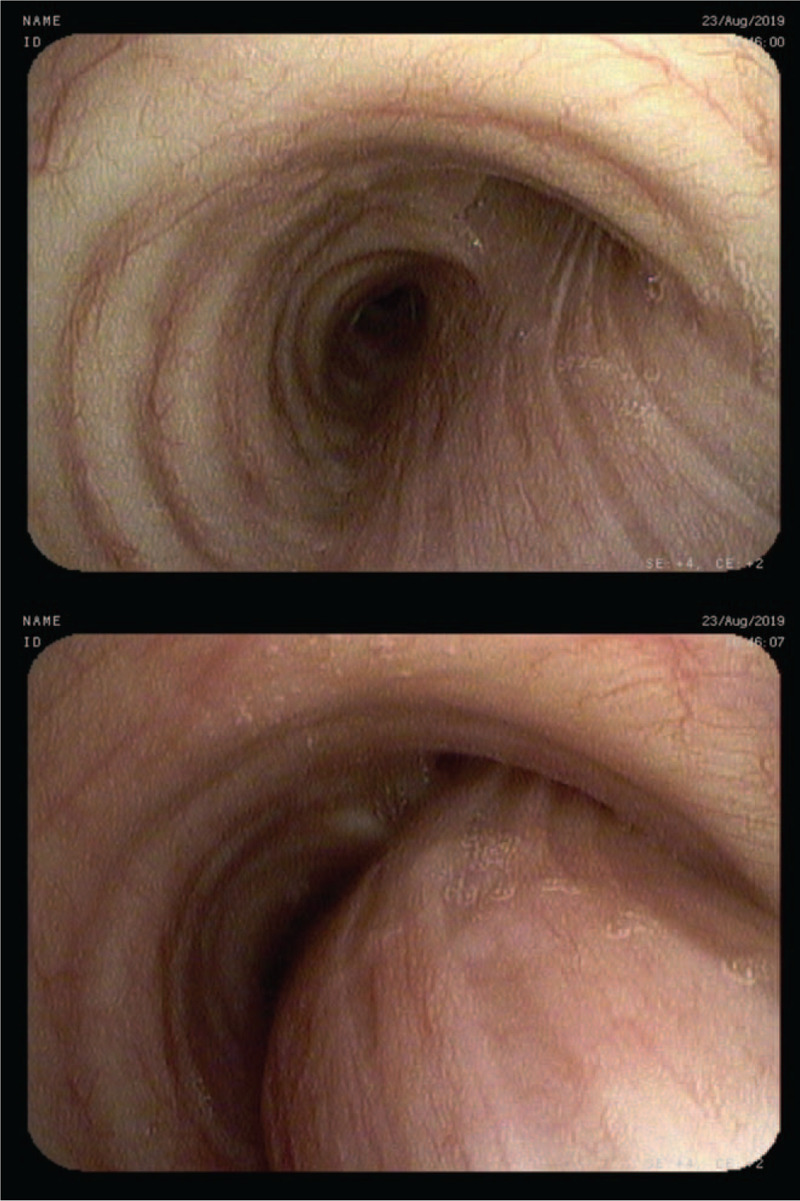
Excess collapse of posterior membrane of the right main bronchus.

**Figure 2:**
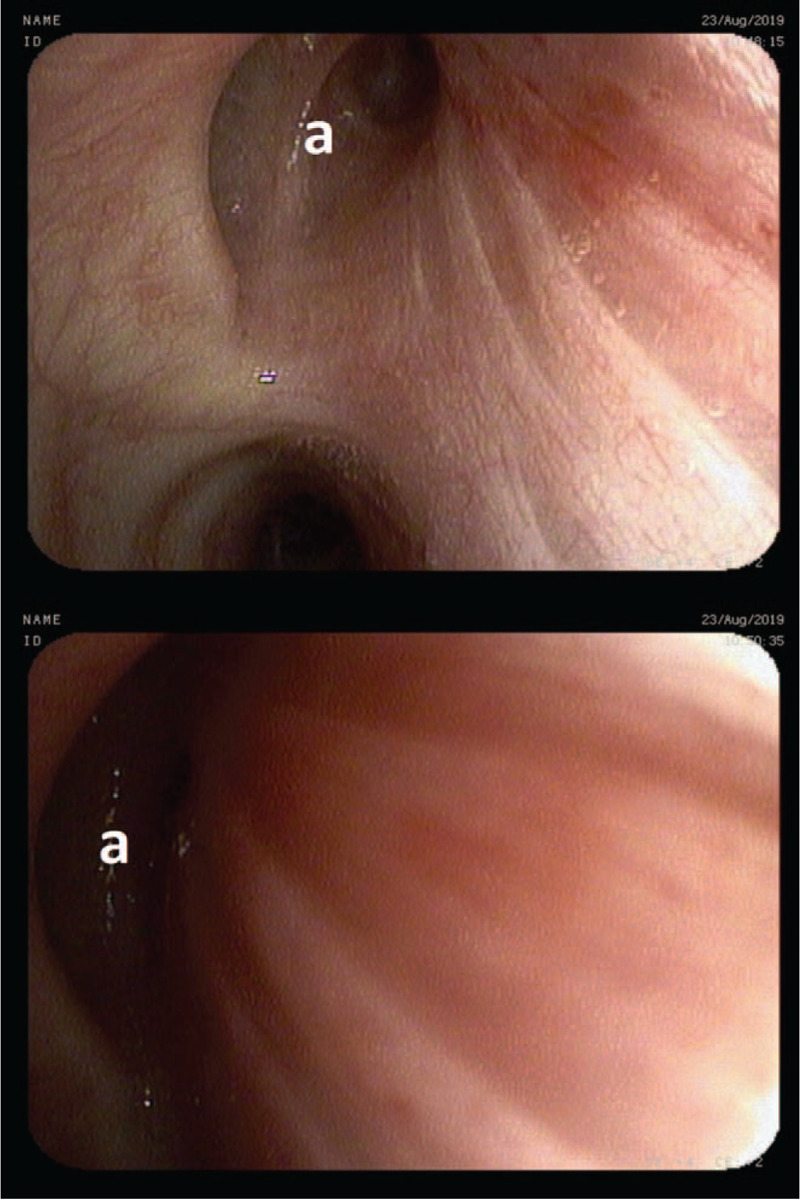
Excess collapse of the right upper lobe bronchus (A).

A 43-year-old non-smoking female was admitted for day-case bronchoscopy because of chronic cough of unknown etiology lasting for 2 years. The cough, accompanied by sputum retention, interfered with daytime activities and subsided at night. Additionally, the patient reported rare heartburns, but denied dyspnea, expiratory wheezing, stridor or hemoptysis. Her medical history included well-controlled hypothyroidism and was negative for allergic diseases, airway hyperreactivity, chronic rhinosinusitis and goiter. Chest CT, otolaryngological examination, echocardiography and gastroscopy provided no clear explanation for chronic cough, trial of inhaled steroids and bronchodilators did not provide its relief. Laboratory results were unremarkable. In multiple outpatient spirometry tests both forced exhalation volume in 1 second (FEV1)%VC and FEV1%FVC were at obstruction threshold. Upright bronchoscopy demonstrated an excess collapse of posterior membrane of right main bronchus (>50%) [Fig. 1] and right upper lobe bronchus (>70%) [Fig. 2] on expiration from tidal to residual volume. Significant amounts of purulent sputum were spotted just behind collapsing part of the right upper lobe bronchus. Sputum cultures, including tuberculosis, were negative. No collapse of other airways was detected using the same maneuvers. The patient was diagnosed as EDAC and discharged home with recommendation of daily N-acetylcysteine and adjustable positive expiratory pressure (PEP) valve usage. She was also referred to an outpatient rehabilitation for assisted cough techniques training. Additionally, owing to the suspicion of laryngopharyngeal reflux, a trial of proton pump inhibitors was recommended. During a follow-up telephone call she reported significant improvement of her symptoms. This was mostly attributed to respiratory physiotherapy employing PEP valve, allowing for increased sputum clearance.

### 1.2 Case #2

**Figure 3:**
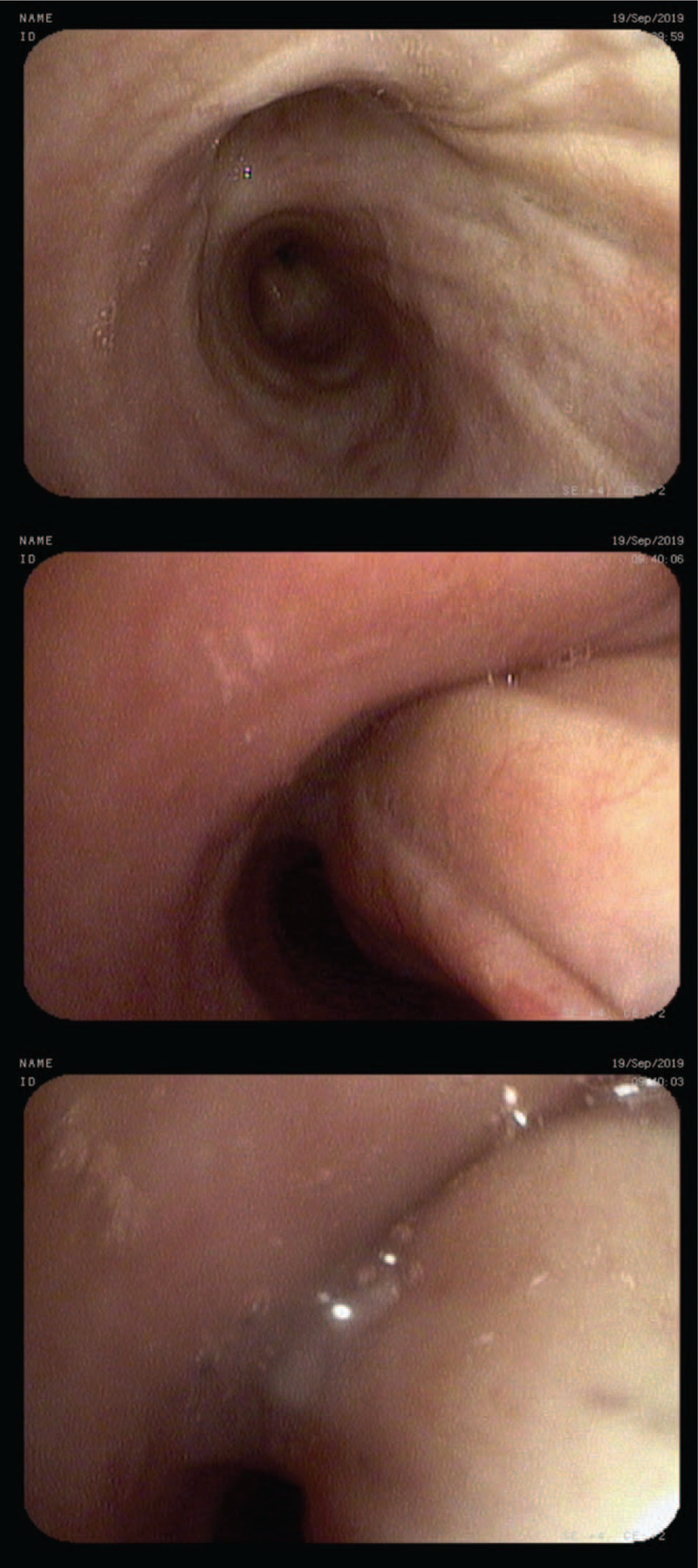
Excess collapse of posterior membrane of right main bronchus and total expiratory collapse of posterior membrane of right upper lobe bronchus.

A 79-year-old obese female was admitted for differential diagnosis of dyspnea of unknown etiology. Dyspnea, present for 7 years, was both exertional and paroxysmal, and was particularly severe in the morning and at night. She additionally reported chronic cough and airway hyperreactivity. These symptoms, accompanied by fever, exacerbated 4 to 5 times a year. A slow relief of these occurrences was achieved by both antibiotics and oral glucocorticoids. Inhaled bronchodilators and corticosteroids used by the patient for the past 7 years did not provide significant relief and the audible wheezing was present continuously. The technique of inhaler usage, assessed on admission, was correct. Before admission she was administered formoterol/beclomethasone metered dose inhaler and oral methylprednisolone 4 mg daily, with ipratropium metered dose inhaler as a rescue therapy. She never smoked, although she was exposed to secondhand smoke during her office work. Her medical history included hiatal hernia. At lung auscultation she presented with significant wheezing, especially dominant over central airways. The laboratory results, apart from steroid-induced leukocytosis, were unremarkable. Ear, nose, and throat examination revealed signs of laryngopharyngeal reflux. Skin prick tests were negative. Spirometry revealed irreversible, mild obstruction, and the body plethysmography a mild air trapping. Chest, high resolution, inspiratory and expiratory, CT confirmed air trapping, small, insignificant bronchiectases in lower lobes, nodules, which were stable compared to earlier outpatient CT image, expiratory collapse of trachea at bifurcation (>50%), and collapse of main bronchi, extent of which could not be precisely measured. During upright bronchoscopy a total collapse of posterior membrane of the right upper lobe bronchus [Fig. 3], and a significant collapse of the right (>70%) [Fig. 3] and left main bronchus (>50%) were detected on expiration from tidal to residual volume. The collapse of a posterior membrane of trachea was normal (<50%). Significant amounts of purulent sputum were collected from beyond the area of excess central airways collapse. Their cultures, including tuberculosis, were negative. No collapse of other airways was detected using the same maneuvers. The patient was diagnosed with EDAC and bronchiectasis overlap and was trained during hospitalization in assisted cough techniques, instructed in gastroesophageal reflux prevention and administered proton pump inhibitors. Other recommendations included daily N-acetylcysteine and adjustable PEP valve usage. During a follow-up telephone call her family reported significant breathlessness reduction attributed to the use of PEP valve and N-acetylcysteine treatment.

## 2 Discussion

In presented cases EDAC was an unexpected finding, even though, it firmly corresponded with reported symptoms. In first case retention of purulent sputum behind collapsing segment of central airway was probably a dominant reason for chronic cough, which might have been exacerbated by gastroesophageal reflux symptoms. In the second case, there was a significant discrepancy between spirometry test and patients respiratory symptoms. These symptoms included paroxysmal dyspnea at night and significant wheezing, loudest over central airways with only a meager improvement after inhaled bronchodilators and glucocorticoids. Dyspnea at night could be partially explained by, postulated by some,^[1,5]^ increased collapse of central airways during sleep in supine position. Ineffectiveness of bronchodilators in ECAC treatment can be explained by their relaxing effect on central airway smooth muscle tone,^[6,7]^ which in turn increases the degree of airway collapse on expiration. In other words, in case of ECAC coexisting with obstructive lung disease, a positive effect of bronchodilators on small airways can be offset by their negative effect on central airway collapse.^[1]^ It is not surprising then, that EDAC is suggested to mimic severe asthma cases,^[8]^ which could be true in patient's #2 case.

Currently, there are no standard criteria for recognizing ECAC.^[1,9]^ Historically, a collapse larger than 50% of cross-sectional area was considered a feature of ECAC,^[1,9]^ however, CT studies on healthy volunteers showed that this threshold might be too low.^[10,11]^ CT studies used also other thresholds than cross-sectional area that is, sagittal and coronal diameters. Those thresholds were measured at different airway levels in different CT modalities that is, end-expiratory or dynamic expiratory,^[1]^ thus rendering their results interpretation and comparison difficult.

There is uncertainty concerning bronchoscopy maneuvers employed to visualize airway collapse^[2]^ and reliability of collapse calculations. This is due to inability of maintaining bronchoscope at the same level of dynamically changing airway when taking pictures or videos.^[5]^

Since incidental detection of airway collapse might not be clinically significant^[12]^ and the degree of collapse considered pathological is unknown, it seems that patient should be diagnosed with ECAC only if characteristic symptoms are present i.e. wheezing on exertion, wheezing dominating over central airways during auscultation, dyspnea refractory to bronchodilator treatment or disproportionate to pulmonary function test results, unexplained paroxysmal dyspnea, recurrent COPD or asthma exacerbations, chronic cough, especially with sputum retention or “seal barking” sound, syncope during cough or forced exhalation,^[2,8]^

Treatment options used in ECAC patients are purely empirical and have not been verified in randomized controlled studies. Nonetheless centers treating ECAC patients^[1,13]^ recommend different respiratory physiotherapy devices including PEP valve, use of expectorants, pulmonary rehabilitation and, in patients with more severe symptoms, continuous positive airway pressure or bilevel positive airway pressure. Carefully selected patients may be considered for stenting or tracheobronchoplasty.^[1]^ Other surgical interventions include tracheostomy, if ECAC is limited to trachea, or resection and reconstruction of affected airway in the case of focal malacia.^[2]^ Taking into consideration that ECAC was shown to be an independent of FEV1, emphysema scores and pack-years risk factor of respiratory exacerbations, both in patients with and without COPD,^[14]^ a question remains whether recommended treatment would be able to reduce their frequency.

## 3 Conclusions

TBM and EDAC, due to their symptoms and lack of awareness amongst clinicians, tend to be mislabeled as common obstructive lung disorders or remain undetected even though they complicate their course. This applies particularly to EDAC, interpreted as a symptom of underlying obstructive lung disease, and not as a separate entity.

## Author contributions

All four authors were engaged in conception and development of the article. Piotr Janowiak prepared the draft of the article whereas remaining three authors were responsible for its critical revision. All authors participated in final approval of the manuscript and agree to be accountable for all aspects of the work.
